# *Salmonella* Proteomic Profiling during Infection Distinguishes the Intracellular Environment of Host Cells

**DOI:** 10.1128/mSystems.00314-18

**Published:** 2019-04-09

**Authors:** Zezhou Li, Yanhua Liu, Jiaqi Fu, Buyu Zhang, Sen Cheng, Mei Wu, Zhen Wang, Jiezhang Jiang, Cheng Chang, Xiaoyun Liu

**Affiliations:** a Institute of Analytical Chemistry and Synthetic and Functional Biomolecules Center, College of Chemistry and Molecular Engineering, Peking University, Beijing, People’s Republic of China; b State Key Laboratory of Proteomics, Beijing Proteome Research Center, Beijing Institute of Lifeomics, National Center for Protein Sciences (Beijing), Beijing, People’s Republic of China; Princeton University

**Keywords:** *Salmonella* proteome, bacterial infection, mass spectrometry, the *his* operon

## Abstract

Essential to bacterial pathogenesis, Salmonella enterica serovar Typhimurium (*S.* Typhimurium) has evolved the capacity to quickly sense and adapt to specific intracellular environment within distinct host cells. Here we examined *S.* Typhimurium proteomic remodeling within macrophages, allowing direct comparison with our previous studies in epithelial cells. In addition to many shared features, our data revealed proteomic signatures highly specific to one type of host cells. Notably, intracellular *S.* Typhimurium differentially regulates the two type III secretion systems (T3SSs) far more quickly in macrophages than in epithelial cells; bacterial flagellar and chemotaxis systems degenerate more quickly in macrophages than in HeLa cells as well. Importantly, our comparative analysis uncovered high levels of induction of bacterial histidine biosynthesis in macrophages but not in epithelial cells. Targeted metabolomic measurements revealed markedly lower histidine levels within macrophages. Intriguingly, further functional studies established that histidine biosynthesis that is defective (due to a *hisG* mutation) renders the bacterium (strain SL1344) hypersensitive to intracellular shortage of this amino acid. Indeed, another *S.* Typhimurium strain, namely, strain 14028s, with a fully functional biosynthetic pathway exhibited only minor induction of the *his* operon within infected macrophages. Our work thus provided novel insights into *S.* Typhimurium adaptation mechanisms within distinct host cells and also provided an elegant paradigm where proteomic profiling of intracellular pathogens is utilized to discriminate specific host environments (e.g., on the basis of nutrient availability).

**IMPORTANCE** *Salmonella* Typhimurium is one of the leading causes of foodborne bacterial infection. Nevertheless, how *Salmonella* adapts to distinct types of host cells during infection remains poorly understood. By contrasting intracellular *Salmonella* proteomes from both infected macrophages and epithelial cells, we found striking proteomic signatures specific to particular types of host cells. Notably, *Salmonella* proteomic remodeling exhibited quicker kinetics in macrophages than in epithelial cells with respect to bacterial virulence and flagellar and chemotaxis systems. Furthermore, we unveiled high levels of induction of bacterial histidine biosynthesis in macrophages but not in epithelial cells, which is attributable to differing intracellular levels of this amino acid. Intriguingly, we found that a defective *hisG* gene renders a *Salmonella* strain hypersensitive to histidine shortage in macrophages. Overall, our work reveals specific *Salmonella* adaptation mechanisms in distinct host cells, which should aid in the development of novel anti-infection strategies.

## Abstract (precis)

*Salmonella* Typhimurium is one of the leading causes of foodborne bacterial infection. Nevertheless, how *Salmonella* adapts to distinct types of host cells during infection remains poorly understood. By contrasting intracellular *Salmonella* proteomes from both infected macrophages and epithelial cells, we found striking proteomic signatures specific to particular types of host cells. Notably, *Salmonella* proteomic remodeling exhibited quicker kinetics in macrophages than in epithelial cells with respect to bacterial virulence and flagellar and chemotaxis systems. Furthermore, we unveiled high levels of induction of bacterial histidine biosynthesis in macrophages but not in epithelial cells, which is attributable to differing intracellular levels of this amino acid. Intriguingly, we found that a defective *hisG* gene renders a *Salmonella* strain hypersensitive to histidine shortage in macrophages. Overall, our work reveals specific *Salmonella* adaptation mechanisms in distinct host cells, which should aid in the development of novel anti-infection strategies.

## INTRODUCTION

As a Gram-negative bacterial pathogen, Salmonella enterica serovar Typhimurium (*S.* Typhimurium) can infect both humans and other animal hosts (1). *Salmonella* infection is usually caused by oral ingestion of contaminated food or water (2). Upon passage/survival through gastric acidity, ingested bacteria can eventually gain access to the intestinal epithelium. Invasion of epithelial cells is facilitated by the injection of a cocktail of virulence factors (called “effectors”) by dedicated *Salmonella* type III secretion systems (T3SSs). *S.* Typhimurium is endowed with two distinct T3SSs encoded on *Salmonella* pathogenicity islands 1 and 2 (SPI-1 and SPI-2), respectively (3). It is thought that the initial bacterial invasion is mediated mostly by the SPI-1 T3SS effectors, whereas those encoded by SPI-2 contribute to the biogenesis of *Salmonella*-containing vacuoles (SCV) (4), thereby promoting intracellular survival and replication.

After crossing the intestinal epithelium, *S.* Typhimurium can be further internalized by phagocytic cells, such as macrophages (5). Macrophages play an important role in host innate immune responses during bacterial infection (6). As professional phagocytes, macrophages have evolved a suite of strategies to eliminate pathogenic bacteria. Two well-characterized pathogen-killing mechanisms depend on the production of an oxidative burst containing reactive oxygen species (ROS) and reactive nitrogen species (RNS) (7). Consequently, *Salmonella* has developed efficient means to detoxify these antimicrobial molecules. Indeed, *Salmonella* survival in macrophages as well as in epithelial cells is essential for bacterial pathogenesis (8). Upon internalization, *Salmonella* must quickly sense and adapt to different intracellular environments (i.e., epithelium or macrophage cells) (9). High-throughput expression profiling of intracellular *Salmonella* by a number of groups, including ours, has contributed significantly to the understanding of bacterial adaptations within infected host cells (10–13).

Here we extended our proteomic profiling of intracellular *S.* Typhimurium from infected epithelial cells to macrophages, allowing the first comparison of bacterial pathogen proteomes within two distinct types of host cells. Despite of many common features, notable differences in the proteomic remodeling of pathogens were observed. Strikingly, the *his* operon of *S.* Typhimurium was highly induced in macrophages whereas the corresponding proteins in infected epithelial cells were barely detected. A number of follow-up functional experiments revealed that such high levels of induction of the *his* operon is attributable to a lower level of histidine within macrophages as well as to the defective biosynthesis of this amino acid in the bacterial strain (SL1344). In fact, *S.* Typhimurium strain 14028s (with functional histidine biosynthesis) was much less sensitive to intracellular limitation of this amino acid. To our knowledge, this work represents the first paradigm of quantitative proteomics of intracellular bacteria distinguishing the host environments of different cell types in terms of nutrient availability.

## RESULTS

### Proteomic landscape of intracellular *S.* Typhimurium within infected macrophages.

**FIG 1:**
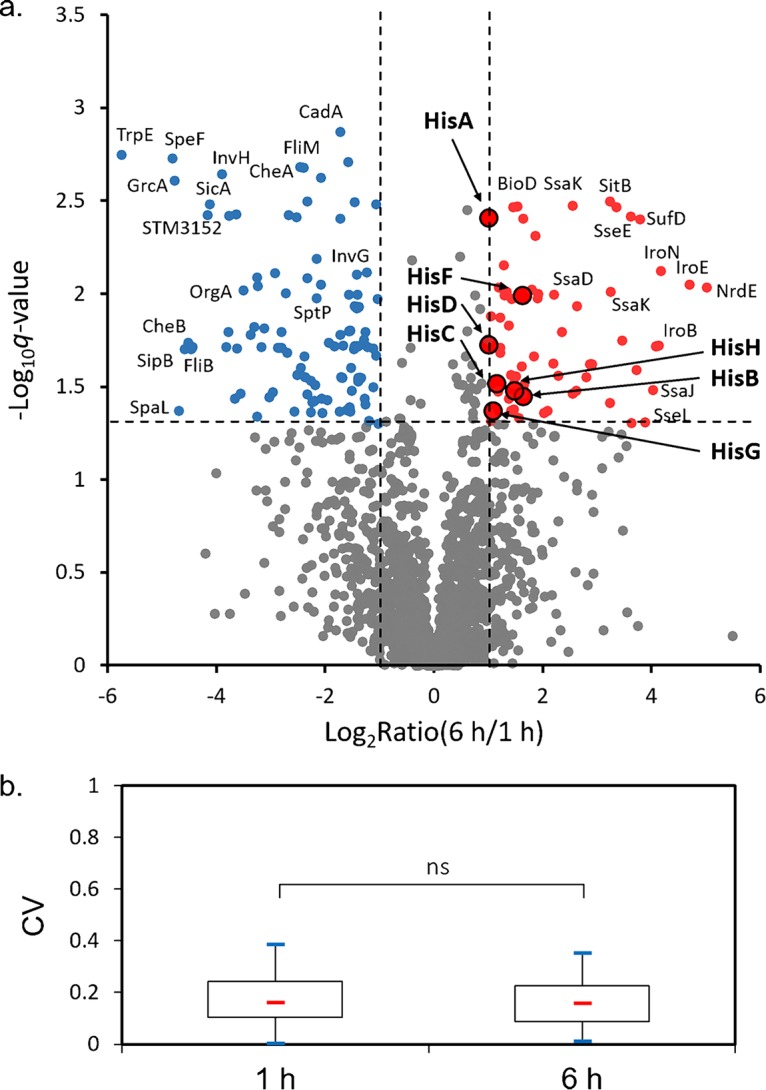
(a) A volcano plot of intracellular *Salmonella* proteins detected by LC-MS/MS in RAW 264.7 cells. The fold change values were calculated by dividing LFQ intensity values measured at 6 hpi by those measured at 1 hpi. The logarithmic ratios of average fold changes are reported on the *x* axis. The *y*-axis data plot negative logarithmic false-discovery-rate (*q*) values from the *t* test performed on three biological replicates. Up- and downregulated proteins are denoted by the red and blue dots, respectively. (b) Box plot visualizing coefficients of variations (CVs) of identified *S.* Typhimurium proteins in three biological replicates at 1 hpi and 6 hpi. The box plot is shown with 25th percentile, median, and 75th percentile values and with whiskers representing minimum and maximum values. Statistical significance was determined by using paired *t* tests (ns, not significant).

To investigate the adaptation strategies employed by *S.* Typhimurium during infection of macrophages, we analyzed the proteome of intracellular pathogens (Salmonella enterica serovar Typhimurium strain SL1344) that were isolated from RAW 264.7 cells at distinct stages of infection (i.e., 1 and 6 h postinfection [hpi]). In total, we identified 1,746 *S.* Typhimurium proteins (covering ∼40% of the bacterial proteome) from three biological replicates, with less than 10% of the identifications resulting from host contaminants (see Table S2 in the supplemental material). As about 86% of *S.* Typhimurium genes were expressed in one environment (14), the actual coverage of our proteomic profiling might be slightly higher. By using label-free quantitation (LFQ), we found 169 differentially expressed bacterial proteins (false-discovery-rate [FDR {*q*}] value of <0.05 and fold change value of >2.0 or <0.5 at 6 hpi relative to 1 hpi) (Table S3). A volcano plot of quantitative proteomic data sets with 69 upregulated proteins (denoted by the red dots) and 100 downregulated proteins (denoted by the blue dots) is shown in Fig. 1a. The biological variations in the normalized LFQ intensities under both sets of experimental conditions (1 and 6 hpi) were visualized with a box plot, with the data indicating good reproducibility of our biological replicates (Fig. 1b).

Among those differentially expressed proteins, many notable features seem to be shared between two types of host cells (RAW 264.7 and HeLa) commonly used for *S.* Typhimurium infection (the intracellular *S.* Typhimurium proteome within HeLa cells was reported in our previous work [12, 13]). For instance, many proteins that are involved in the acquisition of iron (Fe), manganese (Mn), and molybdenum (Mo) were upregulated (2.8-fold to 25.9-fold) at 6 hpi, indicating a general shortage of these metal ions within host cells (Table S3). Another prominent proteomic signature associated with intracellular *S.* Typhimurium is the induction of the SPI-2 T3SS (2.8-fold to 16.2-fold) concomitant with the repression of the SPI-1 T3SS (2.2-26.0-fold) and of the flagellar and chemotaxis systems (2.6-fold to 22.6-fold) at 6 hpi. Indeed, the differential levels of regulation of these two T3SSs were also conserved in both RAW 264.7 and HeLa cells (though occurring at different time points as discussed later). Furthermore, the reprogramming of *S.* Typhimurium metabolic pathways in macrophages closely resembles those proteomic features of intracellular bacteria in HeLa cells (12), such as substantial downregulation of the enzymes in the tricarboxylic acid (TCA) cycle pathway (e.g., SucA, SucC, SucD, FumA, FumB, SdhA, and SdhB) (2.1-fold to 14.2-fold) and the respiratory pathway (both aerobic and anaerobic) (Table S3). Last but not the least, many altered proteins are associated with amino acid metabolism, which also exhibits some features that are common between macrophages and epithelial cells.

Despite the conservation of many proteomic signatures, strikingly, a number of bacterial proteins were found to be exclusively altered in one type of host cells. For instance, all *Salmonella* proteins encoded by the *his* operon, including HisA, HisB, HisC, HisD, HisF, HisH, HisI, and HisG, were markedly upregulated in RAW 264.7 cells. In contrast, these proteins were barely detectable in bacteria isolated from HeLa cells throughout the infection process (12, 13). Moreover, Hmp (2.1-fold) and SodC1 (2.7-fold) were specifically upregulated in RAW 264.7 cells but not in HeLa cells at 6 hpi. Hmp is a bacterial hemoglobin playing a central role in bacterial resistance to reactive nitrogen species (RNS) (15), while SodC1 is able to detoxify superoxide, a common reactive oxygen species (ROS). Therefore, their induction likely protects intracellular *Salmonella* bacteria from the oxidative burst exerted by macrophages. Interestingly, KatG, another antioxidant protein, was also seen at higher levels at 6 hpi in RAW 264.7 than in HeLa cells (Table S4). Together, these findings suggest the presence of both oxidative stress and nitrosative stress in RAW 264.7 macrophages (unlike permissive HeLa cells). On the other hand, exclusive alteration of some proteins was found in HeLa cells (Table S4). For instance, metabolic enzymes in the pentose phosphate pathway (PPP) (e.g., TalA and TktB) and pyrimidine degradation pathway (e.g., DeoA and DeoC) were upregulated in HeLa cells (12) whereas they were not significantly changed in RAW 264.7 cells. We next discuss in greater detail those altered *Salmonella* proteins that exhibited differential patterns between the two types of host cells.

### Faster alteration of *S.* Typhimurium virulence, chemotaxis, and flagellar proteins in macrophage cells.

**FIG 2:**
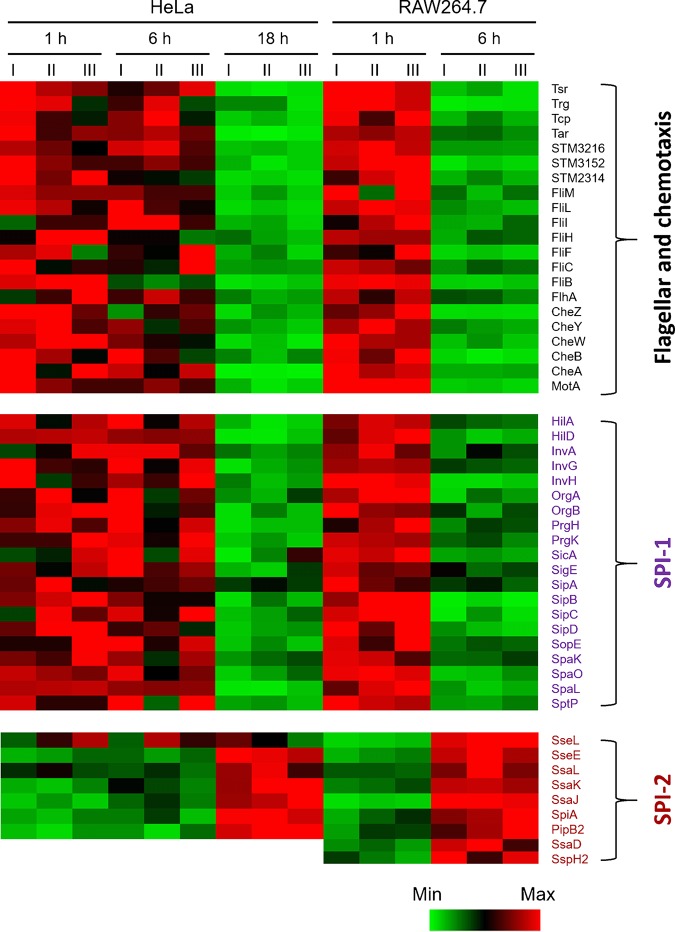
Heat maps showing the abundance levels of a few distinct classes of *Salmonella* proteins detected by LC-MS/MS. Included are SPI-1-encoded and SPI-2-encoded virulence proteins as well as those associated with bacterial flagellar and chemotaxis systems. RAW 264.7 cell data represent expression profiles determined at 1 and 6 hpi; HeLa cell data represent expression profiles determined at 1, 6, and 18 hpi. Each set of columns I to III represents three biological replicates at each sampling time. Min, minimum; Max, maximum.

Among those bacterial products that are differentially regulated in macrophages, many proteins are involved in *S.* Typhimurium virulence, chemotaxis, and flagellar systems. For example, many SPI-2 T3SS-encoded proteins, including SpiA, SsaD, SsaJ, SsaK, SsaL, SseE, SseL, SspH2, and PipB2, were markedly induced (2.8-fold to 16.2-fold) at 6 hpi. The upregulation of SPI-2 proteins was often accompanied by repression of SPI-1 T3SS. Indeed, we found significant downregulation of 20 virulence factors associated with SPI-1 T3SS. These findings are in general consistent with the well-documented SPI-1 suppression and SPI-2 induction seen upon *S.* Typhimurium internalization into host cells (4). Nevertheless, striking differences were observed in the kinetics of these proteomic alterations between the two cell types. In macrophages, the expression of both T3SSs was substantially altered at 6 hpi, whereas similar proteomic reprogramming was much delayed during *S.* Typhimurium infection of epithelial cells. In HeLa cells, while some slight differences may be discernible at 6 hpi, it was not until 18 hpi that differential regulation of two T3SSs was observed at a magnitude comparable to that seen in RAW 264.7 macrophages (Fig. 2). For instance, in RAW 264.7 cells, SPI-1-encoded SipC was 8-fold less abundant at 6 hpi. At that stage, in contrast, no significant change was seen in HeLa cells, though the levels seen with those cells were decreased by 6-fold at 18 hpi. Furthermore, a SPI-2 protein, SseL, was specifically induced in RAW 264.7 cells but not in HeLa cells in our experiments.

Another major proteomic signature of intracellular *S.* Typhimurium is the degeneration of bacterial flagellar and chemotaxis systems. Intriguingly, the repression of these bacterial components also features quicker kinetics in macrophages than in epithelial cells (Fig. 2). While evident only at 18 hpi in HeLa cells, substantial repression of flagellar and chemotaxis proteins was observed much earlier (at 6 hpi) in RAW 264.7 cells. The flagellin protein FliC, for example, was already downregulated by 3-fold at 6 hpi in RAW 264.7 cells, whereas its repression was delayed to 18 hpi (4-fold decrease) in epithelial cells. In addition, many flagellar proteins (FliB, FliL, FliF, and FliM) (9.0-fold to 22.6-fold change) and chemotaxis proteins (CheZ, CheY, CheB, Tsr, and Trg) (7.0-fold to 21.8-fold change) were more severely downregulated at 6 hpi in RAW 264.7 than in HeLa cells (13). Taken together, these findings further reinforce the notion of a nonmotile state of intracellular *S.* Typhimurium and, importantly, that such adaptations occur much earlier in macrophages than in epithelial cells.

### Massive induction of *S.* Typhimurium histidine biosynthesis pathway in RAW 264.7 cells but not in HeLa cells.

**FIG 3:**
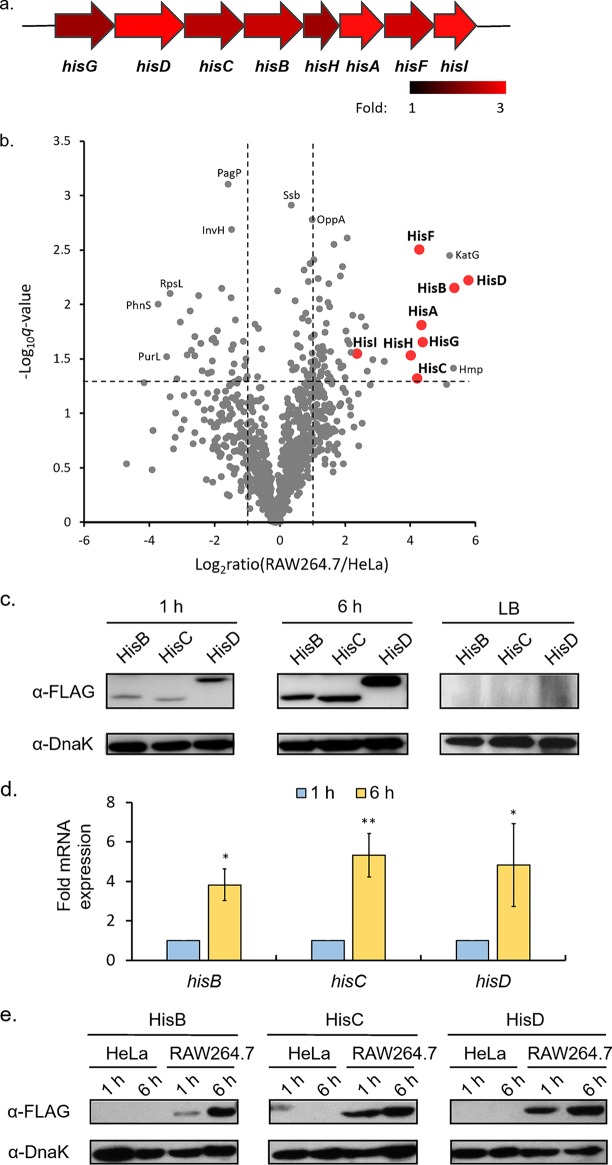
(a) A schematic diagram of the *his* operon showing the fold changes of corresponding proteins in RAW 264.7 cells at 6 hpi compared to 1 hpi. (b) A volcano plot of intracellular *Salmonella* proteins detected by LC-MS/MS within two types of host cells. The fold change values were calculated by dividing LFQ intensity values determined at 6 hpi in RAW 264.7 cells by those determined at the same time point in HeLa cells. The logarithmic ratios of average fold changes are reported on the *x* axis. The *y* axis plots negative logarithmic *q* values from the *t* test performed on three biological replicates. (c) Western blot analyses of representative *Salmonella* His enzymes at 1 and 6 hpi in RAW 264.7 cells or in LB media. (d) qRT-PCR analyses of mRNA samples extracted from intracellular *Salmonella* at 6 hpi relative to 1 hpi (*n *=* *3). Statistical significance was determined by using paired *t* tests (*, *P* < 0.05; **, *P* < 0.01). (e) Western blot analyses of representative *Salmonella* His enzymes performed at 1 and 6 hpi in HeLa and RAW 264.7 cells.

Perhaps the most striking difference between the two types of host cells with respect to the intracellular *S.* Typhimurium proteomes is the exclusive induction of the *his* operon in RAW 264.7 cells. In total, this operon encodes eight enzymes responsible for unbranched histidine biosynthesis, yielding l-histidine from phosphoribosyl pyrophosphate (PRPP). Indeed, all of the His enzymes had higher expression levels in RAW 264.7 cells at 6 hpi than at 1 hpi (Fig. 3a). To directly compare *S.* Typhimurium protein abundances within different host cells, we further plotted the ratios of bacterial protein intensities in RAW 264.7 cells to those in HeLa cells at 6 hpi (Fig. 3b) (More details about the comparisons can be found in Table S4). Notably, all His proteins were dispersed to the right upper corner of the volcano plot, suggesting that the abundances of these bacterial enzymes differed most between macrophages and HeLa cells. It is also interesting that these histidine biosynthesis proteins were less prominent in the previous volcano plot, where protein intensities in macrophages were compared between 1 hpi and 6 hpi (Fig. 1a). One possible explanation is that the His enzymes were already induced to higher levels at as early as 1 hpi in macrophages. Consistent with this hypothesis, our previous study showed that these enzymes were barely expressed in extracellular bacteria grown *in vitro* (13). Furthermore, their expression within epithelial host cells remained consistently low at basal levels throughout the infection process (12, 13).

To further confirm these proteomic changes, we next constructed *S.* Typhimurium strains chromosomally expressing 3×FLAG-tagged His enzymes (HisB-FLAG, HisC-FLAG, and HisD-FLAG) and assayed their expression levels during infection of RAW 264.7 cells. Immunoblotting analyses clearly demonstrated markedly increased levels of HisB, HisC, and HisD in macrophages at 6 hpi compared to 1 hpi (Fig. 3c), consistent with our data from liquid chromatography-mass spectrometry (LC-MS)-based label-free protein quantification. Under similar assay conditions, these three enzymes were barely expressed in *S.* Typhimurium cultured in LB media (Fig. 3c, right panel). Furthermore, we extracted bacterial RNA from the same set of intracellular *S.* Typhimurium samples; reverse transcription-quantitative PCR (qRT-PCR) analyses demonstrated the induction of the corresponding *his* genes on the transcript level as well (Fig. 3d).

We next sought to further validate the specificity of His upregulation in one type of host cells but not in the other. At both 1 hpi and 6 hpi, intracellular bacteria were isolated from infected RAW 264.7 or HeLa cells. As the rates of *S.* Typhimurium multiplication differ among different host cells (16), we adjusted sample loading such that similar amounts of intracellular bacteria were used for immunoblotting analyses (Fig. 3e). As previously shown, HisB, HisC, and HisD were readily detected at 1 hpi in infected macrophages and their levels were found to have increased robustly by 6 hpi. In contrast, these His enzymes were barely detected during infection of epithelial cells at either time point, consistent with our previous proteomic data. Taken together, these findings established the specific induction of the *S.* Typhimurium histidine biosynthesis pathway in infected RAW 264.7 cells but not in HeLa cells.

### Intracellular histidine shortage in macrophages contributed to the induction of *S.* Typhimurium *his* operon.

**FIG 4:**
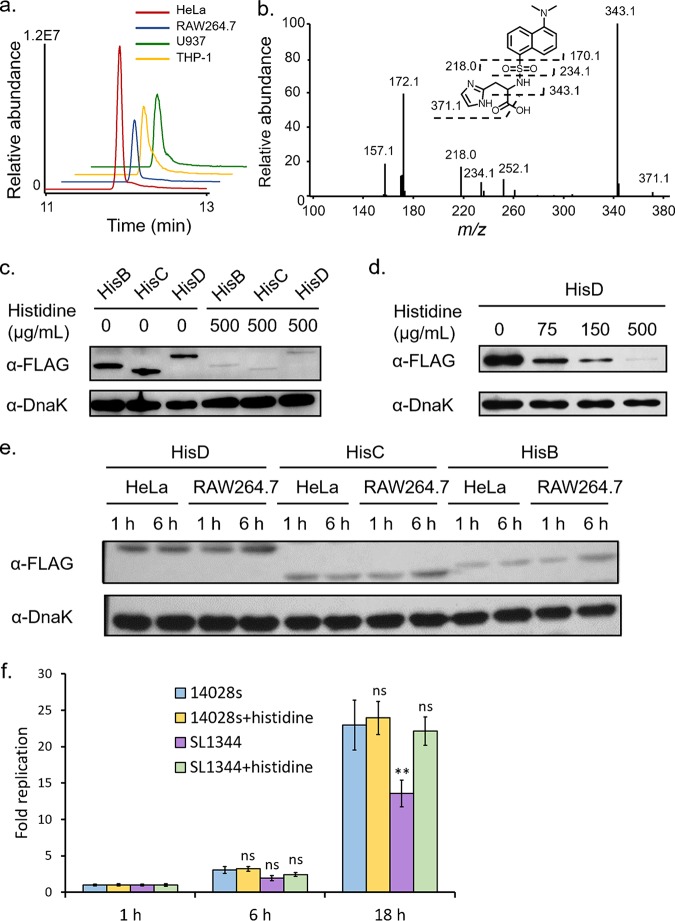
(a) Targeted analyses of histidine levels in HeLa and RAW 264.7 cells performed with the following MS/MS transition: *m/z* 389.12 → 343.12. (b) MS/MS analysis of protonated dansyl histidine. (c) Western blot analyses of representative *Salmonella* His enzymes at 6 hpi in RAW 264.7 cells that were cultured in either regular DMEM or DMEM containing additional histidine (500 μg/ml). (d) Dose-dependent blockage of HisD induction by histidine supplementation in the culture medium. (e) Western blot analyses of representative *Salmonella* 14028s His enzymes at 1 and 6 hpi in HeLa and RAW 264.7 cells. (f) Intracellular growth of *Salmonella* strains SL1344 and 14028s in RAW 264.7 cells that were cultured in either regular DMEM or DMEM with additional histidine (500 μg/ml). Statistical significance was determined by using paired *t* tests (**, *P* < 0.01; ns, not significant).

We reasoned that the specific upregulation of the *his* operon in macrophages was likely due to the low concentration of intracellular histidine. To test this possibility, we set out to determine the levels of bulk intracellular histidine within those two types of host cells by targeted LC-MS experiments as the measurement of the vacuolar histidine level is technically challenging. To facilitate the retention of this amino acid on reversed-phase columns and also to enhance electrospray ionization signals, we employed a dansyl chloride-based precolumn derivatization method (17). High-resolution MS and tandem MS (MS/MS) analyses confirmed the successful formation of dansyl histidine upon performance of derivatization reactions (Fig. 4b). Intracellular metabolites were extracted from host cell pellets (about 50 μl), and the derivatized samples were subjected to targeted LC-MS analyses. As shown in Fig. 4a, the level of histidine was markedly lower in RAW 264.7 cells than in HeLa cells. By using the volume of cell pellets as a proxy for that of cells, we in fact determined the concentration of intracellular histidine in HeLa cells as approximately 0.2 mM, whereas in RAW 264.7 cells it was only 0.06 mM. Furthermore, it is rather interesting that the LC-MS analyses also suggested the presence of lower histidine levels in two human macrophage cell lines (U937 and THP-1) than in HeLa cells (Fig. 4a). These data suggest that the induction of the *his* operon was likely due to the lower histidine level in macrophages than in HeLa cells.

To further test this hypothesis, we next sought to administrate extracellular histidine to replenish its intracellular pool during bacterial infection of macrophages. Indeed, when 500 μg/ml histidine was added to the culture medium, immunoblotting assays barely detected *S.* Typhimurium HisB, HisC, and HisD proteins at 6 hpi (Fig. 4c), suggesting that these enzymes were no longer induced upon bacterial internalization. Furthermore, we explored the dose-dependent impact of extracellular histidine on the expression of histidine biosynthetic enzymes (i.e., HisD). Immunoblotting data demonstrated that the expression levels of HisD at 6 hpi decreased with increasing concentrations of extracellular histidine in the culture medium (Fig. 4d). Notably, administration of 75 μg/ml histidine was able to prevent the induction of HisD within macrophages. Therefore, these findings suggest that intracellular scarcity of histidine indeed contributes to the induction of the *his* operon upon bacterial uptake into macrophages.

### Defective *S.* Typhimurium histidine biosynthesis was critical to the high levels of induction of the *his* operon.

Though we found marked differences in histidine levels between the two cell types, the extent of such differences is far lower than that of the induction of the His enzymes. Previous studies suggested that *S.* Typhimurium strain SL1344 is auxotrophic for histidine because HisG is not functional due to a missense mutation (*hisG46*) (18, 19). As a result, this strain has lost its ability to synthesize histidine, which we reasoned might have led to exceedingly high levels of His enzymes. To test this possibility, we utilized *S.* Typhimurium strain 14028s, which has a functional histidine biosynthesis pathway, for the infection assays. In fact, immunoblotting analyses showed very minor (and yet discernible) induction of His enzymes within infected RAW 264.7 cells at 6 hpi (Fig. 4e). For *S.* Typhimurium 14028s isolated from HeLa cells, these bacterial enzymes were consistently at low levels during the course of infection, similar to what was previously shown for strain SL1344. Together, these findings suggest that both an intracellular histidine shortage and defective histidine biosynthesis are necessary to promote high levels of induction of the *his* operon in *S.* Typhimurium SL1344, a histidine auxotrophy, within infected macrophages.

Given the essential role of histidine in supporting bacterial growth and the differences between *S.* Typhimurium strains SL1344 and 14028s, we next assayed their intracellular replication within infected RAW 264.7 cells, where the histidine supply is more limited than in HeLa cells. At 18 hpi, *S.* Typhimurium strain 14028s clearly outgrew SL1344, though such differences were not pronounced at 6 hpi (Fig. 4f). Furthermore, supplementation of extracellular histidine restored the intracellular growth of *S.* Typhimurium SL1344 to a level comparable to that of 14028s (Fig. 4f), whereas addition of extra histidine did not enhance the replication of 14028s. These data suggest that deficient histidine biosynthesis in SL1344 may have contributed to its retarded growth within macrophage cells. Consistent with our data, Henry et al. reported similar findings regarding the replication of these two strains within infected MelJuSo cells (19).

## DISCUSSION

The pathogenicity of *S.* Typhimurium relies on its ability to invade and replicate within both nonphagocytic epithelial cells and phagocytic cells such as macrophages (5, 20). Therefore, *S.* Typhimurium must quickly adapt to specific intracellular environments within different types of host cells. Though a number of nutritional and environmental cues sensed by intracellular *Salmonella* have been uncovered, how environmental specificities in distinct host cells fine-tune bacterial protein expression remains undetermined. Previously, we characterized the *S.* Typhimurium intracellular proteome within infected epithelial cells, revealing extensive bacterial adaptations and the scarcity of a number of nutrients in host cytosol. Our current study focused on similarities and differences of *S.* Typhimurium expression profiles inside macrophages. In total, we detected 1,746 bacterial proteins in isolated *S.* Typhimurium from infected RAW 264.7 cells (versus the 315 proteins reported previously [21]).

Overall, many proteomic features of *S.* Typhimurium are shared between two types of host cells, suggesting the intracellular presence of common environmental cues. For instance, we observed the induction of metal ion transportation/utilization pathways and repression of TCA and anaerobic respiration pathways in both RAW 264.7 and HeLa cells. Nonetheless, our data also revealed host-specific patterns of bacterial protein expression. Notably, several intracellular *S.* Typhimurium adaptation processes exhibit faster kinetics in macrophages. For example, SPI-1 repression and SPI-2 induction occurred much earlier in RAW 264.7 cells than in HeLa cells. Given the major role of SPI-1 T3SS in bacterial invasion, its quicker suppression in RAW 264.7 cells seems to be consistent with the phagocytic nature of macrophages, where *S.* Typhimurium can be internalized in SPI1-independent processes such as macropinocytosis (22). Previous studies have shown that both the onset of *S.* Typhimurium replication and the formation of *Salmonella*-induced filaments (Sifs) are delayed in macrophages (4), which seems to be counterintuitive with respect to the quicker induction of SPI-2 T3SS. Nevertheless, given the coordinated regulation of two T3SSs (23), it is tempting to speculate that the earlier induction of SPI-2 may, at least in part, be a result of quicker downregulation of SPI-1 T3SS. In addition to different kinetics in the regulation of bacterial virulence, our data also revealed quicker degeneration of *S.* Typhimurium flagellar and chemotaxis systems in macrophages than in epithelial cells, though the specific environmental cues leading to such differences remain to be determined. It is also important to note that delayed alteration of these *S.* Typhimurium proteins (i.e., T3SSs, flagellum, and chemotaxis) in HeLa cells is not due to retarded growth of intracellular bacteria. In fact, in our hands, slightly higher rates of *S.* Typhimurium replication were observed within HeLa cells than within macrophages. Rather, such differences in protein regulation of intracellular bacteria are more likely due to specific environmental cues within distinct types of host cells.

A unique proteomic signature of intracellular *S.* Typhimurium inside RAW 264.7 cells is that of the high levels of induction of histidine biosynthesis pathway, suggesting an intracellular shortage of this amino acid in macrophages. Strikingly, all the gene products encoded by the *his* operon were concordantly upregulated in our proteomic data set, which was further verified by both immunoblotting and mRNA measurements. Previously, it was shown that certain nutrients such as aromatic amino acids and purine bases are limiting inside host cells (18, 24), a finding that is supported by the intracellular growth defects of corresponding auxotrophic strains. Nevertheless, the biochemical makeup of mammalian cells has not been defined in detail (25). Unlike results seen with other nutrients, histidine deficiency seems to be exclusive to RAW 264.7 cells and not HeLa cells, leading to the induction of the *his* operon only in macrophages. Several lines of evidence seem to favor this hypothesis as follows. (i) Administration of extracellular histidine blocks the induction of the *his* operon in a dose-dependent manner. (ii) Histidine supplementation promotes *S.* Typhimurium replication inside macrophages. (iii) Targeted LC-MS measurements indicate a three-times-lower level of histidine inside macrophages than inside HeLa cells.

**FIG 5:**
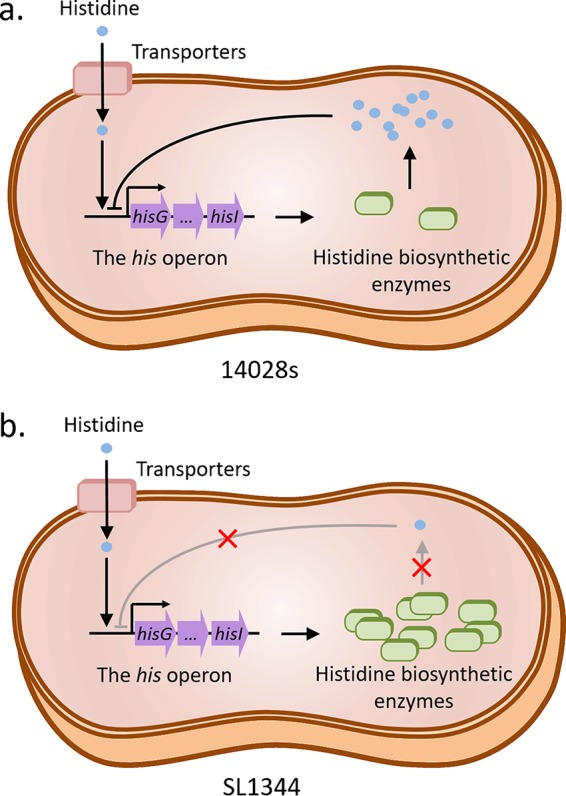
A proposed model for distinct processes of regulation of histidine biosynthesis in two *Salmonella* strains during infection of macrophage cells. Unlike strain 14028s (a), *hisG* mutation in strain SL1344 (b) results in defective histidine biosynthesis and the lack of a negative-feedback loop, which leads to enormously high levels of expression of the *his* operon.

Though the hypothesis presented above is attractive, reconciliation of the immense differences (comprising at least an order of magnitude for some proteins) in the levels of His enzymes with the 3-fold difference in histidine levels within the two types of host cells seems rather difficult. In other words, differing histidine levels inside host cells can account only partially for the high levels of variation in the expression levels of His enzymes. Given the fact that strain SL1344 is auxotrophic for histidine (due to HisG mutation), we reasoned that defective histidine biosynthesis could contribute as well to the massive induction of the *his* operon within macrophages. Consistent with this notion, when host cells were infected by another *S.* Typhimurium strain (14028s) capable of histidine biosynthesis, we did not observe substantial upregulation of His enzymes in either macrophages or epithelial cells. Furthermore, histidine supplementation did not boost the intracellular growth of this strain within macrophages. Taken together, these findings seem to support the following model (Fig. 5): upon bacterial internalization into macrophages, the lower level of intracellular histidine serves as an environmental cue to first induce the expression of the histidine biosynthesis pathway (and yet to do so to a minor extent). In case of strain 14028s, synthesis of more histidine would replenish its intracellular pool, which in turn would negatively regulate the expression of His enzymes (Fig. 5a). And yet for a histidine autotrophy strain such as SL1344, mild induction of histidine biosynthesis cannot eliminate an intracellular shortage of this amino acid. This sustaining demand in turn serves as an additional cue for further induction of the *his* operon, eventually leading to uncontrolled, high expression levels (Fig. 5b). In other words, the high level of upregulation can be attributed to the lack of a negative-feedback mechanism in SL1344, which is required to maintain cellular homeostasis of histidine biosynthesis.

In summary, our comparative analyses of *S.* Typhimurium proteome within distinct types of host cells revealed many conserved features as well as hallmarks unique to specific intracellular environments. Notably, our comprehensive profiling data serve to distinguish the bacterial intracellular niche inside macrophages from that in epithelial cells by the differing levels of a single amino acid (histidine). Of note, data from LC-MS analyses of two human macrophage cell lines also suggested the presence of lower levels of intracellular histidine than in HeLa cells. Further studies in more-diverse types of cell lines would be desirable to test the extensiveness of our findings. Nonetheless, it is tempting to speculate that macrophage cells may have evolved a mechanism maintaining low levels of certain nutrients (i.e., histidine) to restrict proliferation of intracellular bacterial pathogens, which is reminiscent of the use of iron deprivation as a defense strategy by many host cells. Furthermore, our proteomic profiling strategies can be broadly utilized to probe the subtle differences in the intracellular environments within mammalian cells, which are often not readily accessed by conventional approaches.

## MATERIALS AND METHODS

### Bacterial strains, cultivation, and molecular cloning.

Two Salmonella enterica serovar Typhimurium strains (SL1344 and 14028s) were used in this study. All the proteomic experiments were carried out with strain SL1344, whereas both strains were usable in the other experiments. All bacterial strains were stored under −80°C conditions in 25% (vol/vol) glycerol. The frozen bacteria were first streaked and cultivated on LB agar plates overnight at 37°C. A single colony was picked and inoculated into 3 ml of LB medium. The next morning, the culture was further diluted 1:20 into fresh LB medium and was grown at 37°C until mid-exponential phase (optical density at 600 nm [OD_600_] of 0.9). *Salmonella* mutants and strains chromosomally expressing 3×FLAG-tagged proteins were constructed by using a lambda red recombination system as previously described (26). For the tagging of chromosomal genes, the sequence encoding the 3×FLAG epitope was inserted in-frame at the C terminus of the gene of interest. All primers and strains used in this study are listed in Table S1 in the supplemental material.

10.1128/mSystems.00314-18.1 TABLE S1 List of all primers and strains used in the study. Download Table S1, XLS file, 0.03 MB. Copyright © 2019 Li et al. 2019 Li et al. This content is distributed under the terms of the Creative Commons Attribution 4.0 International license.

10.1128/mSystems.00314-18.2 TABLE S2 *Salmonella* proteins detected by LC-MS/MS as well as host contaminants. (All LFQ intensity values were derived from raw data.) Download Table S2, XLS file, 0.5 MB. Copyright © 2019 Li et al. 2019 Li et al. This content is distributed under the terms of the Creative Commons Attribution 4.0 International license.

10.1128/mSystems.00314-18.3 TABLE S3 *Salmonella* proteins whose levels exhibited significant changes at 6 h postinfection (relative to 1 hpi). The displayed numbers represent log_2_ (LFQ intensity), and missing intensity values were replaced with random numbers from a normal distribution. Download Table S3, XLS file, 0.10 MB. Copyright © 2019 Li et al. 2019 Li et al. This content is distributed under the terms of the Creative Commons Attribution 4.0 International license.

10.1128/mSystems.00314-18.4 TABLE S4 Comparison of intracellular *Salmonella* proteins levels at 6 h postinfection in infected RAW 264.7 and HeLa cells. Download Table S4, XLS file, 0.3 MB. Copyright © 2019 Li et al. 2019 Li et al. This content is distributed under the terms of the Creative Commons Attribution 4.0 International license.

### Mammalian cell culturing, bacterial infection, and isolation of intracellular *Salmonella*.

HeLa cells, murine macrophage cell line RAW 264.7, and two human macrophage cell lines (U937 and THP-1) were cultured in Dulbecco’s modified Eagle medium (DMEM; HyClone, USA) supplemented with 10% fetal bovine serum (FBS; PAN-Biotech, Germany) under 5% CO_2_ conditions at 37°C. Human macrophage cell lines were used only for the purpose of determining intracellular histidine levels, and RAW 264.7 cells were used for all relevant experiments. Before infection, mid-exponential-phase (OD_600_ = 0.9) bacteria in LB medium (with 0.3 M NaCl) were pelleted by centrifugation at 3,000 × *g* and then resuspended with an equal volume of prewarmed (37°C) Hanks’ buffered salt solution (HBSS, HyClone, US). *Salmonella* infection was carried out in HBSS with a multiplicity of infection (MOI) of 100 for 30 min. Subsequently, cell monolayers were washed three times with prewarmed (37°C) HBSS and incubated in prewarmed DMEM with 100 μg/ml gentamicin for 1 h to kill extracellular bacteria. The culture medium was further changed to DMEM with 10 μg/ml gentamicin. At 1 and 6 h postinfection (hpi; timed from the addition of DMEM with 100 μg/ml gentamicin), cells were washed three times with phosphate-buffered saline (PBS) and then lysed by the use of 20 mM Tris-HCl buffer (pH 7.6) containing 150 mM NaCl and 0.1% Triton X-100. Cell lysates were first centrifuged at 600 × *g* for 5 min to remove nuclei and cell debris. The resulting fractions were further spun at 4,000 × *g* for 20 min to pellet intracellular bacteria. Harvested bacterial pellets were washed with radioimmunoprecipitation assay (RIPA) buffer (25 mM Tris-HCl [pH 7.6], 150 mM NaCl, 1% Triton X-100, 0.5% sodium deoxycholate, 0.1% SDS) to remove residual host contaminants. Finally, bacterial samples were resuspended in the SDS-PAGE sample buffer and heated at 95°C for 5 min prior to gel fractionation. For *Salmonella* intracellular growth assays, RAW 264.7 cells were seeded in 6-well plates and the infection was carried out at an MOI of 1. At the indicated time points, infected cells were lysed and viable intracellular bacteria were numerated by CFU assays.

### Proteomic analyses of intracellular bacteria.

Bacterial samples isolated from RAW 264.7 cells were fractionated by using 10% SDS-PAGE, processed into 8 gel bands, and subjected to in-gel trypsin digestion as previously described (27). Peptides were extracted, vacuum dried, and resuspended in solvent A (97% H_2_O, 3% acetonitrile [ACN], 0.1% formic acid [FA]) for proteomic analyses. Liquid chromatography-tandem mass spectrometry (LC-MS/MS) experiments were performed on a hybrid ion trap-Orbitrap mass spectrometer (LTQ-Orbitrap Velos; Thermo Scientific) coupled with nanoflow reversed-phase liquid chromatography (EASY-nLC 1000; Thermo Scientific). The capillary column (75 μm by 150 mm) was packed with 4-μm-diameter, 100-Å Magic C18AQ silica-based particles (Michrom BioResources Inc., Auburn, CA) and was equipped with a laser-pulled electrospray tip (Model P-2000; Sutter Instruments). A 40-min gradient was employed that ranged from 7% to 35% solvent B (100% ACN, 0.1% FA). Eluted peptides were electrosprayed directly into the mass spectrometer for MS and MS/MS analyses in a data-dependent acquisition mode. The 10 ions with the highest intensity from the full MS scan (*m/z* 350 to 1,500) were selected for MS/MS analyses. Dynamic exclusion was set with a repeat duration of 24 s and an exclusion duration of 12 s. Three biological replicates of intracellular *Salmonella* samples were analyzed.

### Experimental design and statistical rationale.

To investigate the adaptation strategies that *S.* Typhimurium employed during infection of macrophages, we analyzed the proteome of intracellular bacteria isolated from RAW 264.7 cells at 6 hpi in comparison to those isolated at 1 hpi. In total, we analyzed three biological replicates in 48 LC-MS/MS experiments. MaxQuant (http://maxquant.org/; version 1.5.4.1) was used to generate peak lists from raw MS files, and Andromeda was used for database searching with the following parameters: oxidation (M) as a variable modification, precursor mass tolerance at 20 ppm, trypsin as the enzyme, fragment mass tolerance at 0.8 Da, and maximum of two missed cleavages. Acquired MS/MS spectra were searched against a merged database that includes the Mus musculus protein database (version 2016_11) and an S. enterica serovar Typhimurium LT2 protein database complemented with 191 unique proteins from strain SL1344 (LT2 is far better annotated than SL1344, with 4,533 sequences downloaded from UniProt, version 2016_11). The false-discovery rates (FDR) for peptides and proteins were controlled at <1%. MaxQuant software was used to calculate the normalized label-free quantitation (LFQ) intensity for each protein (28). Only the proteins that were detected in ≥2 individual samples from either the 1-hpi group or the 6-hpi group and that had at least two unique peptides were selected as quantified proteins. We removed only those protein hits that were assigned with modifications or that matched entries in the reverse database as well as common contaminants. Logarithmic values (Log_2_) of LFQ intensity were further processed using Perseus software (version 1.5.4.1), and the missing values were replaced with random numbers from a normal distribution (width = 0.3, shift = 1.8). The *t* test statistics were applied with a Benjamini-Hochberg-based FDR value of 5% (*q* value of <0.05) for multiple-hypothesis testing. Proteins with *q* values of <0.05 and ratios (6 hpi/1 hpi) of >2.0 or <0.5 were considered differentially expressed between 6 hpi and 1 hpi.

### Western blotting analyses.

*Salmonella* strains chromosomally producing 3×FLAG-tagged proteins were used for bacterial infection, and intracellular bacteria were isolated at the indicated times. Bacterial pellets were resuspended in the SDS loading buffer. After SDS-PAGE separation, bacterial proteins were transferred to polyvinylidene diﬂuoride (PVDF) membranes. Individual samples were probed with primary antibodies speciﬁc for FLAG (Sigma-Aldrich, F3165) (1:2,000) or *Salmonella* DnaK (Abcam, ab69617) (1:5,000) and horseradish peroxidase (HRP)-conjugated secondary antibodies (Sigma-Aldrich, A4416) (1:5,000).

### Quantitative real-time PCR.

RAW 264.7 cells were infected by *Salmonella*, and intracellular bacteria were isolated as described above. Total RNA of the intracellular bacteria was extracted by using an EasyPure RNA kit (TransGen Biotech, China) and then treated with DNase I to remove DNA. Reverse transcription of RNA was performed with TransScript One-Step genomic DNA (gDNA) removal and cDNA synthesis SuperMix (TransGen Biotech, China). Reverse transcription-PCR (RT-PCR) analyses were carried out on an Applied Biosystems Vii 7 real-time PCR system by using UltraSYBR mixture (low ROX) (CWBio, China). To quantitatively compare the levels of *hisB*, *hisC*, and *hisD* transcripts, the 16S rRNA housekeeping gene was used for normalization. The mRNA levels were determined using the comparative threshold cycle number (2^−ΔΔ^*^CT^*) method (29).

### LC-MS quantification of histidine levels in host cells.

The cell monolayers were thoroughly washed with 5 ml of PBS three times to remove extracellular histidine before harvesting. Cell pellets were lysed in 1 ml of 90% methanol for 15 min followed by centrifugation at 10,000 × *g* for 5 min. The histidine-containing supernatants were vacuum dried. Histidine derivatization was performed as described previously (17). Briefly, samples were resuspended in 50 μl of 0.3 M Na_2_CO_3_ buffer (pH 9.5). After the addition of 50 μl of 5 mg/liter dansyl chloride solution, the reaction mixtures were incubated in a 60°C water bath for 50 min. Subsequently, 30 μl of 0.5 M butyl amine was added to the mixtures prior to further incubation at 60°C for 30 min to consume redundant dansyl chloride. After derivatization, samples were vacuum dried and resuspended in 1 ml of solvent A. For LC-MS analyses, 5 μl of each sample was injected. A 20-min gradient was employed that ranged from 7% to 90% solvent B during 10 min and was then maintained for 10 min before 100% solvent A was used for column equilibration. Eluted molecules were measured by selective reaction monitoring (SRM) with the following transition: *m/z* 389.12 → 343.12.

### Data availability.

The proteomics data reported in this paper have been deposited in the open-access database iProx and are available under accession number IPX0001304001.
